# B cell epitope of human cytomegalovirus phosphoprotein 65 (HCMV pp65) induced anti-dsDNA antibody in BALB/c mice

**DOI:** 10.1186/s13075-017-1268-2

**Published:** 2017-03-21

**Authors:** Ao HoHsieh, Chin Man Wang, Yeong-Jian Jan Wu, Albert Chen, Ming-I Chang, Ji-Yih Chen

**Affiliations:** 1 Department of Medicine, Division of Allergy, Immunology and Rheumatology, Chang Gung Memorial Hospital, Taoyuan, Taiwan, Republic of China; 2 Department of Rehabilitation, Chang Gung Memorial Hospital, Chang Gung University College of Medicine, Taoyuan, Taiwan, Republic of China; 3 Department of Medicine, Division of Allergy, Immunology and Rheumatology, Chang Gung Memorial Hospital, Chang Gung University College of Medicine, No. 5, Fu-Shin St. Kwei-Shan, Taoyuan, 33375 Taiwan, Republic of China; 4 Department of Radiation Oncology, Baylor College of Medicine, Houston, TX USA; 5 Biologics, Ruen Huei Biopharmaceuticals, 1F, No.16-1, Ln. 119, Sec. 1, Roosevelt Rd., Jhongjheng Dist., Taipei, Taiwan, Republic of China

**Keywords:** Systemic lupus erythematosus, Human cytomegalovirus phosphoprotein 65, Glomerulonephritis, Anti-dsDNA antibody

## Abstract

**Background:**

HCMV phosphoprotein 65 (HCMVpp65) is a putative immunogen that acts as an accelerator, inducing autoantibody and exacerbating autoimmune response in susceptible animals. The immunity to pp65_336-439_ instigates autoimmunity, suggesting that pp65_336-439_ contains crucial B cell epitope(s) for the development of nephritis. This study narrowed down the target epitope to pp65_422-439_ for immunization of BALB/c mice and mapping of B cell epitope.

**Methods:**

The target epitope pp65_422-439_ reactivity and B cell epitope mapping was examined in serum from pp65_422-439_-immunized mice and patients with systemic lupus erythematosus (SLE). Kidney tissue from immunized mice was examined for signs of immune complex nephritis.

**Results:**

Anti-pp65_422-439_ antibody in serum either from patients with SLE or from pp65_422-439_-immunized mice exhibited cross-reactivity to several nuclear components such as double-stranded DNA (dsDNA). Moreover, the pp65_422-439_-immunized mice developed initial signs of glomerulonephritis such as deposition of immunoglobulin G/M (IgG/IgM) and third complement component (C3). With B cell epitope mapping by pp65_422-439_-derived decapeptides, one dominant epitope, pp65_428-437_, was identified in serum from pp65_422-439_-immunized mice and patients with SLE with anti-pp65_422-439_ antibody. Epitope spreading from pp65_428-437_ to pp65_430-439_ was found in pp65_422-439_-immunized mice in which we generated monoclonal antibodies to pp65_425-434_ and pp65_430-439_. However, dsDNA positive reactivity was exclusively observed in *Crithidia luciliae* stains with pp65_430-439_-reactive monoclonal antibody. Additionally, we observed the amelioration of autoimmunity following the elevation of IgM targeting pp65_428-437._

**Conclusions:**

Our data suggest that pp65_428-437_ may be an autoimmune or lupus-prone B cell epitope and may catalyze further epitope spreading for inducing autoantibodies in lupus-susceptible individuals.

**Electronic supplementary material:**

The online version of this article (doi:10.1186/s13075-017-1268-2) contains supplementary material, which is available to authorized users.

## Background

Systemic lupus erythematous (SLE) is a chronic autoimmune disease characterized by widespread loss of immune tolerance to self-antigens. Pathogen recognition and subsequent immune responses are potentially the important initiators of autoimmunity in genetically predisposed persons. Emerging evidence indicates that in patients with lupus, exposure to human cytomegalovirus (HCMV) or Epstein-Barr virus (EBV), often precedes the onset of tolerance break [1–3]. EBV is the most studied example for cross-reactive autoantibody-mediated autoimmunity. Cross-reactivity of anti-Epstein Barr virus antigen-1 (EBNA-1) antibody to Ro or spliceosomal proteins has been reported [4–6]. Anti-Sm antibody has been found to cross-react in EBNA-1-immunized animals, underlying the molecular mimicry between these antigens [7–10].

HCMV, a ubiquitous opportunistic pathogen, induces 60 kD/Ro expression on the surface of human keratinocytes [11]. Immunization of lupus-prone mice by HCMV recombinant glycoprotein B (gB) results in the production of significant autoantibody to the U1-70 kDa spliceosome protein [12]. Also, the significant correlation between antibody to HCMV and U1 small nuclear ribonucleoprotein (snRNP) in HCMV-infected patients with SLE implies that HCMV infection is associated with the development of SLE [13]. In addition, immunization of BALB/c mice with a surrogate octapeptide, DWEYSVWLSN, which induces anti-dsDNA antibody, suggests that the shared structural similarity of antigenic determinants among pathogens and self-proteins leads to autoantibody production [14]. The DNA-interacting amino acids of necrotic cells from post-infected hosts may contribute to induction of anti-dsDNA antibodies [15].

HCMV phosphoprotein 65 (pp65) is a viral scaffold protein and the most abundant constituent of the extracellular viral particle [16]. The pp65 is involved in modulating viral kinase activity and attenuating host antiviral responses [17, 18]. The pp65 protein is a target of both cellular and humoral immunity in healthy individuals, but dominant T cell epitope(s) leads to the robust cellular responses such as cytotoxic T lymphocyte response [19, 20]. Highly elevated anti-pp65 titers in patients with SLE and immunization of NZB/W F1 mice by pp65 induces early onset of lupus-like symptoms, implying a potential role of pp65 in SLE [21].

The immunization of truncated pp65_336-439_-conjugated C3d has been shown to induce lupus-like autoantibodies and subsequent development of autoimmunity [22]. The current study aims to further identify the autoantibody-inducing B cell epitope(s) within pp65_386-439_ and the potential pathogenic immune response.

## Methods

### Characteristics of the study populations

All patients were recruited from the clinics of Chang Gung Memorial Hospital, and rheumatology specialists confirmed that all patients fulfilled the 1982 and 1997 American College of Rheumatology (ACR) diagnostic criteria for SLE [23, 24] This study was approved by the Institutional Review Board of Chang Gung Medical Foundation. The study of methods was carried out in accordance with the relevant guidelines and informed consent was obtained from all subjects.

### Mice

Normal female BALB/c mice, 3–5 weeks old, were purchased from the National Laboratory Animal Center (NLAC), Taiwan. Animals were housed in a pathogen-free facility with an independent ventilation cage system at the laboratory animal center of Chang Gung Memorial Hospital. All BALB/c mice were 8 weeks old at inoculation.

### Synthetic peptides

For all synthetic peptides, the purity of the peptide was >95%, per the peptide manufacturer (GenScript, NJ, USA). The preparation of peptides followed the manufacturer’s instructions (20 μg/μl), with storage at -80 °C prior to use. Six histidines and one cysteine were added at the C terminus of the peptide as a target or for crosslinking to a carrier protein via a disulfide bond.

### Plasmid construction

The full-length pp65 sequence was amplified from pCMV6-pp65 (SKU VC101263, Origene, FJ527563) using the following paired primers (forward 5′GCGGATATCATGGAGAGCCGGGGCCGG, reverse 5′ GCGGGATCCGCCTCTATGCTTCTTGGG). The pp65 sequence was prepared from PCR and digested by EcoRV/BamHI, then ligated into pET30. The murine C3d encoding sequence (GenBank: DQ408205) was PCR-amplified with C3d primers (forward 5′CGCGGATCCATGACCCCCGCAGGCTGTGGG, reverse 5′CGCGCTCGAGGCTACGGCTGGGGAGGTG) and ligated into pET30.

### Antigen preparation

The C3d biotinylation (Pierce, Thermo Scientific, IL, USA) and streptavidin (SA) (Pierce) conjugation were performed as per the manufacturers’ protocol. In brief, maleimide-activated streptavidin (Pierce) was conjugated with peptide containing reduced disulfide bonds from a disulfide reducing gel (Pierce) and mixed with biotinylated C3d to form the peptide-SA-biotin-C3d tetramer, including pp65_386-403_, pp65_422-439_ and SA-C3d only. Tetramers were generated and prepared for immunization within 4 hours.

### Immunization and serum collection

Female BALB/c mice (n = 28) were randomly separated into groups receiving pp65_386-403_- (n = 9), pp65_422-439_-C3d (n = 9), SA-C3d (n = 5) or PBS (n = 5). Mice were inoculated subcutaneously with 100 μg (2 μg/μl) pp65_386-403_-C3d, pp65_422-439_-C3d, SA-C3d or 50 μl PBS in complete Freund’s adjuvant (CFA, Sigma Aldrich, MO, USA) at day 0, respectively. Boosting was performed with antigens in incomplete Freund’s adjuvant (IFA, Sigma Aldrich) at day 14, day 28 and day 42. Mice were bled via the retro orbital vein one day prior to each assay and at 2-week intervals. Unused serum was stored at -80 °C and the PBS-diluted seruma was kept at 4 °C.

### Antibody preparation, biotinylation and streptavidin conjugation

Recombinant proteins were over-expressed in *Escherichia coli* with 1 mM isopropyl β-D-thiogalactoside induction (IPTG, Sigma Aldrich) and purified by a nickel affinity column (Sigma Aldrich). Antibody preparation was performed as previously described [22]. In brief, moderated cyanogen bromide (CnBr) powder (Sigma Aldrich) was activated following the manufacturer’s protocol. A total of 2 mg of four tandem repeats of the pp65_422-439_ peptides (GGGSGGGAMAGASTSAGRKRKS) was dissolved by gentle rotation in a coupling buffer (0.1 M NaHCO_3_, 0.5 M NaCl, pH 8.3) with activated CnBr gel at 4 °C overnight. The free active groups on CnBr were deactivated by 0.1 M Tris-HCl (pH 8.0) at room temperature (RT) for 2 hours. After deactivation, CnBr gel was washed with alternating buffer (0.1 M NaAc, 0.5 M NaCl, pH 4.0 and 0.1 M Tris-HCl, 0.5 M NaCl, pH 8.0) twice and washed with 10 ml PBS once. For purification, 10 ml of serum from twenty dsDNA-positive or dsDNA-negative patients with SLE with pp65_422-439_ antibody in 20 ml PBS, respectively, were added to pp65_422-439-_conjugated CnBr gel and rolled at 4 °C overnight. The flow-through was collected and concentrated as a negative control, while bound antibodies were eluted by 1 ml of 0.1 M glycine (pH 2.0). The eluted samples were neutralized immediately with 30 μl of neutralizing buffer (1 M Tris-HCl, 2 M NaCl, pH 8.8).

### Enzyme-linked immunosorbent assay (ELISA)

ELISA was performed as previous described [22]. Briefly, for the anti-pp65 peptide (pp65_386-439_, pp65_386-403_, pp65_396-413_, pp65_404-421_, pp65_414-431_, pp65_422-439_ and nine pp65_422-439_-derived decapeptides) or anti-dsDNA antibody assay, 1 μg/well of synthetic peptide or purified calf thymus dsDNA (Sigma Aldrich) in coating buffer (150 mM Na_2_CO_3_, 150 mM NaHCO_3_, pH 9.6) was coated to a microtiter 96-well plate (Greiner Bio-One, CA, USA) at 4 °C overnight. After blocking with 5% skimmed milk, 250× diluted human or mice serum, 3 μg purified pp65_422-439_ antibody or 1 μg monoclonal antibodies in PBS were added and incubated at 37 °C for 2 hours.

For the competitive inhibition assay, anti-pp65_422-439_ purified antibody was co-incubated with 1 μg pp65_422-439_ or dsDNA in 200 μl PBS at RT for one hour. The mixture was transferred to one well of a 96-well plate coated with dsDNA or pp65_422-439_ for incubation at 37 °C for 2 hours. At the end of the incubation, the microtiter plate was washed four times with PBST (PBS with 0.05% Tween 20) and bound antibody was detected by horseradish peroxidase (HRP)-conjugated anti-human/mouse G/M or anti-mouse IgG subclasses (IgG1, IgG2a, IgG2b and IgG3) at a dilution of 1:5000 (Jackson ImmunoResearch Laboratories, PA, USA) at 37 °C for 2 hours. For detection of cross-reactivity to host proteins, 1 μg/well of homogenized HEK293T cell lysate was coated on a microtiter plate at 4 °C overnight. After blocking, mice serum was diluted and bound antibodies were detected as described above. O-phenylenediamine dihydrochloride (OPD, Sigma Aldrich) was used as the substrate in ELISA buffer (250 mM Na_2_HPO_4_, 175 mM C_6_H_8_O_7_, pH 5.0) and HRP activity was read at 450 nm with a micro ELISA reader (Molecular Devices).

### Western blot/slot blot

Full-length pp65 protein (40 μg/per gel) was separated by 12% SDS-PAGE (slab gel format). Separated protein was transferred to nitrocellulose paper, blocked by 5% skimmed milk and then analyzed with 1 μg/ml anti-His-tag antibody (eBioscience, CA. USA), 100× diluted human sera or 3 μg purified pp65_422-439_ antibody in PBS at RT for 2 hours. Antibody reactivity was detected by HRP-conjugated secondary antibody (Jackson ImmunoResearch Laboratories) and chemiluminescent detection reagent (Millipore, MA. USA).

### Anti-nuclear antibodies, *C. luciliae* and kidney immunofluorescence stain

Mouse serum was tested for anti-nuclear antibodies (ANAs) at 1:100 dilutions in PBS using a standard anti-nuclear antibody (ANA) test (Diasorin, Saluggia, Italy). The reactivity of anti-dsDNA antibody was examined by immunofluorescence stain using the *C. luciliae* test (Diasorin) at dilutions of 1:20, 1:40 and 1:80 in PBS, as per manufacturer’s instruction. In brief, 30 μl of diluted mice serum, 3 μg purified pp65_422-439_ antibody or 1 μg monoclonal antibodies were incubated on a slide coated with HEp-2 or *C. luciliae* at RT for 30 minutes in a humidified chamber. Slides were washed three times in 50 ml PBS at RT for 10 minutes each.

Bound antibodies were detected by 100× diluted FITC-conjugated anti-mouse IgG/M (Jackson ImmunoResearch Laboratories) at RT for 30 minutes in a darkened and humidified chamber. For nuclear visualization, the HEp-2 slide was incubated in 30 μl of 4',6-diamidino-2-phenylindole (DAPI) (1 mg/ml, Sigma Aldrich) at RT for 5 minutes in the dark. At the end of staining, slides were washed with PBS for 30 seconds and mounted via mounting medium (Diasorin) for investigation by fluorescence microscopy (Olympus DP72). For immunofluorescence staining of the glomerulus, kidneys were removed from the mice, immediately placed in optimal cutting temperature (OCT) gel and frozen at -80 °C for 24 hours. The 5-μm-thick frozen sections were stained with FITC-conjugated anti-mouse IgM/G (Jackson ImmunoResearch Laboratories) at a 1:100 dilution in PBS at RT for 30 minutes in a humidified chamber in the dark. After PBS washing, coverslips with mounting medium (Diasorin) on tissue slides were prepared for investigation by fluorescence microscopy.

### Hybridoma preparation

The hybridoma was prepared following the manufacturer’s instructions (Roche, Basel, Switzerland) with minor modifications. Briefly, the mouse spleen cells were mixed at a ratio to Sp2/0-Ag14 of 5:1 (ATCC, VA, USA) in a sterile 50-ml conical tube, which was centrifuged to pellet the cells at 800 rpm for 10 minutes. After discarding the supernatant, 1 ml of 50% PEG 1500 (Roche) was slowly added to the cell pellet dropwise over a 1-minute period and the cells were swirled for 90 seconds in a 37 °C water bath. Cell fusion was stopped by adding Roswell Park Memorial Institute medium (RPMI) 1640 (Gibco, CA, USA) containing 10% fetal bovine serum (FBS, Invitrogen, CA, USA) with gentle swirling at RT for 10 minutes. After washing with RPMI 1640 twice, cells were suspended in 30 ml of RPMI1640 supplemented with 10% FBS, 10% BM Condimed H1 (Roche) and 1x HAT (Gibco), plated 2.5 ml per well in a 6-well culture dish and incubated at 37 °C in a 5% CO_2_ incubator. Limiting dilution was carried out for selection of a single colony, which was amplified in RPMI 1640 supplemented with 10% FBS, 10% BM Condimed H1 (Roche), 1× HT (Gibco) and 1x hybridoma fusion & cloning supplement (Roche). The supernatant was harvested for ELISA of antibody activity to pp65_422-439_.

### Statistical analysis

Statistical differences in titer and prevalence were analyzed using GraphPad Prism (GraphPad Software Inc.). The Student *t* test, two-tailed Fisher’s test, and Mann-Whitney test were used for these comparisons with graphs depicting mean ± SEM. A 5% level of significance for *p* values was used for all analyses.

## Results

### Elevated anti-pp65_422-439_ reactivity related to dsDNA positivity in SLE

The fragment of pp66_336-439_ induced autoantibodies and immunoglobulin (Ig) deposition on glomeruli in BALB/c mice has been reported [22]. Due to a poor humoral response to pp65_336-385_ in patients with SLE, we examined anti-pp65_386-439_ reactivity to reveal the critical B cell epitopes using serum from 238 patients with SLE (119 with and 119 without anti-dsDNA reactivity), 86 patients with ankylosing spondylitis (AS), 78 patients with rheumatoid arthritis (RA) and 84 healthy controls. As shown in Table 1, 83 of 238 patients with SLE (34.87%) had higher incidence of antibody reactivity to pp65_386-439_ compared to the 1/86 patients with AS (1.16%), 4/78 patients with RA (5.28%) or 1/84 normal controls (1.20%). Of the 119 Patients with SLE with anti-dsDNA positive serum (termed SLE-dsDNA(+)), 52/119 patients were also positive for anti-pp65_386-439_ activity (43.70%). On the other hand, 31/119 patients with SLE with anti-dsDNA negative sera (termed as SLE-dsDNA(-)) (26.05%) were anti-pp65_386-439_ positive. Human serum positive for pp65_386-439_ was reconfirmed by western blotting (Additional file 1: Figure S1).

**Table 1:** The prevalence of antibody to HCMVpp65_386-439_ in patients with autoimmunity and healthy controls

		SLE-dsDNA(+)	SLE-dsDNA(-)	AS	RA	Normal
Age (years)		16-77	22-75	15-66	20-89	32-64
Mean (years)		39.5	35.2	37.1	56.2	43.2
Total specimen		119	119	86	78	84
Female (%)		100	100	18	82	100
Responsiveness	pp65_386 to 439_ (%)	52/119	31/119	1/86 (1.16)	4/78 (5.13)	1/84 (1.19)
		(43.70)	(26.05)			

RA: rheumatoid arthritis; AS: ankylosing spondylitis

**Fig. 1:**
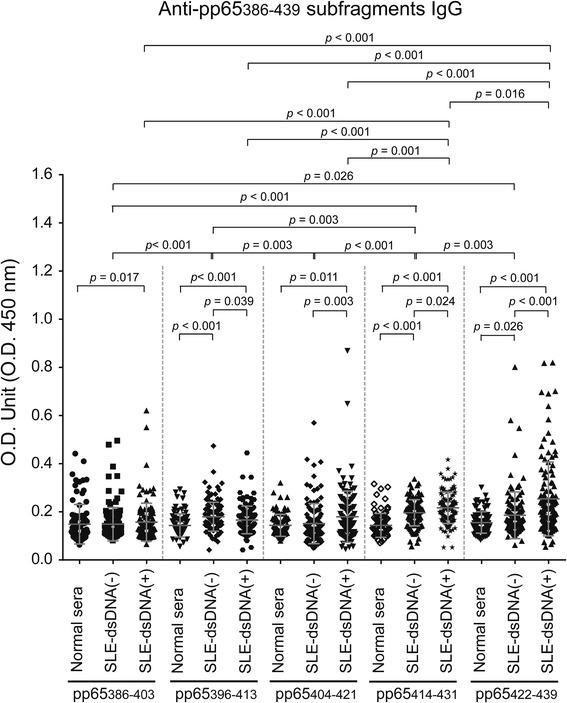
Detection of IgG antibody against pp65_422-439_ subfragments by serum from patients with systemic lupus erythematosus (*SLE*) and healthy controls. ELISA was performed for IgG against five pp65 subfragments, pp65_386-403_, pp65_396-413_, pp65_404-421_, pp65_414-431_ and pp65_422-439_, using serum from patients with SLE with or without anti-double-stranded DNA antibody (*SLE-dsDNA*(+), n = 119; SLE-dsDNA(-), n = 119) and normal controls (n = 84). 250× diluted sera were used. Data are presented as the mean ± SEM of three independent experiments. *O.D.* optical density

Next, we synthesized pp65_386-403_, pp65_404-421_ and pp65_422-439_, covering the entire pp65_386-439_, and re-screened serum from patients with SLE and healthy controls using ELISA to identify the dominant epitope(s). As shown in Fig. 1, in the SLE subgroups there was significant elevation of IgG antibody to pp65_422-439_ (SLE-dsDNA(+) 0.254 ± 0.014, *p* < 0.001; SLE-dsDNA(-), 0.186 ± 0.009, *p* = 0.026) and pp65_404-421_ (SLE-dsDNA(+), 0.180 ± 0.011, *p* = 0.004; SLE-dsDNA(-), 0.151 ± 0.008) compared to normal controls (0.156 ± 0.009; 0.144 ± 0.005). The IgG antibody titer for pp65_422-439_ was greater than that for pp65_404-421_ in the SLE subgroups (SLE-dsDNA(+), *p <* 0.001; SLE-dsDNA(-), *p* = 0.003). Moreover, SLE-dsDNA(+) had greater anti-pp65_422-439_ reactivity than SLE-dsDNA(-) (*p* < 0.001).

In further epitopes analysis, antibody against pp65_422-439_ was significantly elevated in patients with SLE-dsDNA(+) compared to pp65_396-413_ (0.163 ± 0.005, *p* < 0.001) or pp65_414-431_ (0.180 ± 0.01, *p* = 0.016). On the other hand, we were unable to purify anti-pp65_422-439_ antibody using CnBr conjugated with four tandem repeated pp65_422-439_ peptide from disease or healthy controls, suggesting that the lower titer of anti-pp65_422-439_ antibody is unavailable for purification. Together, these findings suggested anti-pp65_422-439_ reactivity is specific to patients with SLE and related to dsDNA positivity.

### Anti-pp65_422-439_ antibody showed cross-reactivity to nuclear proteins and dsDNA

**Fig. 2:**
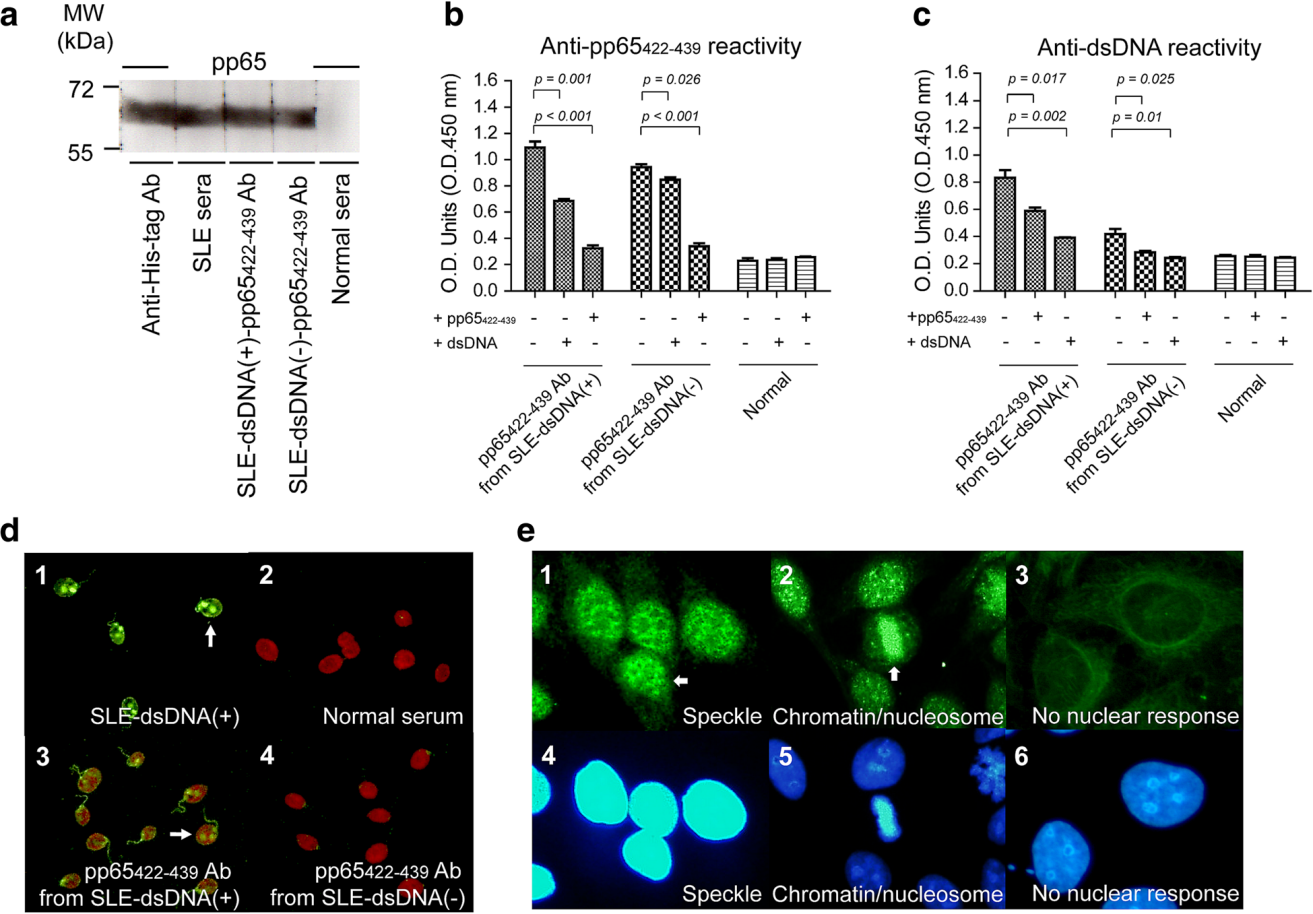
Detection of cross-reactivity in affinity-purified pp65_422-439_-specific IgG in serum from patients with systemic lupus erythematosus (*SLE*). **a** Immunoblot analysis with serum from patients with SLE, healthy controls and anti-pp65_422-439_ antibody from ten pooled SLE-double-stranded DNA (*SLE-dsDNA*(+) or SLE-dsDNA(-)) serum against human cytomegalovirus (HCMV)pp65. We used 100× diluted serum from patients with SLE or healthy controls and anti-His-tag antibody as positive and negative controls. Molecular mass markers (kD) are shown on the left. *MW* molecular weight, *kDa* kilodalton. **b**-**c** ELISA for anti-pp65_422-439_ and anti-dsDNA activity: 100× diluted normal serum and 3 μg anti-pp65_422-439_ antibody from SLE-dsDNA(+) or SLE-dsDNA(-) sera were used. For the competitive inhibitory assay, 1 μg/well of pp65_422-439_ or dsDNA was used. **d** Representatives of *C. luciliae* stain by 100× diluted serum from SLE-dsDNA(+) (*d1*) or healthy control and anti-pp65_422-439_ antibody (*d2*) from SLE-dsDNA(+) (*d3*) or SLE-dsDNA(-) (*d4*) serum. *White arrowheads* indicate the positive stains. **e** HEp-2 substrate slides were used for detection of anti-nuclear antibodies. Patterns of speckle (*e4*) and nucleosome/chromatin (*e2*, *e5*) stains were revealed by anti-pp65_422-439_ antibody from SLE-dsDNA(+) or SLE-dsDNA(-) serum. Nuclear reactivity was not observed from 100× diluted normal serum (*e3*, *e6*). *White arrowheads* indicate the patterns of nuclear response. These results are representative of triplicated experiments. *O.D.* optical density

To elucidate the relationship between pp65_422-439_ and autoantibodies developed in patients with SLE, antibodies to pp65_422-439_ were affinity-purified in pooled serum from SLE-dsDNA(+) or SLE-dsDNA(-). The anti-pp65_422-439_ antibodies from both SLE subgroups exhibited anti-pp65 activities (Fig. 2a). The purified anti-pp65_422-439_ antibodies could be inhibited by pp65_422-439_ or partially inhibited by dsDNA in both SLE groups (Fig. 2b). Also, there was cross-reactivity of anti-pp65_422-439_ antibody to dsNDA suppressed by pp65_422-439_ or dsDNA in the two SLE groups (Fig. 2c). Notably, the titer of anti-dsDNA antibody was significantly higher in SLE-dsDNA(+) (0.833 ± 0.056) than in SLE-dsDNA(-) (0.418 ± 0.037, *p* = 0.004; Fig. 2c). However, the anti-dsDNA activity was solely exhibited by *C. luciliae* stain with anti-pp65_422-439_ antibody from SLE-dsDNA(+) (Fig. 2d). Indirect immunofluorescence stain on purified serum from patients with SLE revealed clear speckle stains (Figs. 2e1, e4). Notably, stain from nucleosome/chromatin is unique to pp65_422-439_ specific antibody from SLE-dsDNA(+) (Fig. 2e2, e5). In contrast, no nuclear responses were observed in normal serum stain (Fig. 2e3, e6).

### The pp65_422-439_ immunization induced cross-reactive antibodies to nuclear components

**Fig. 3:**
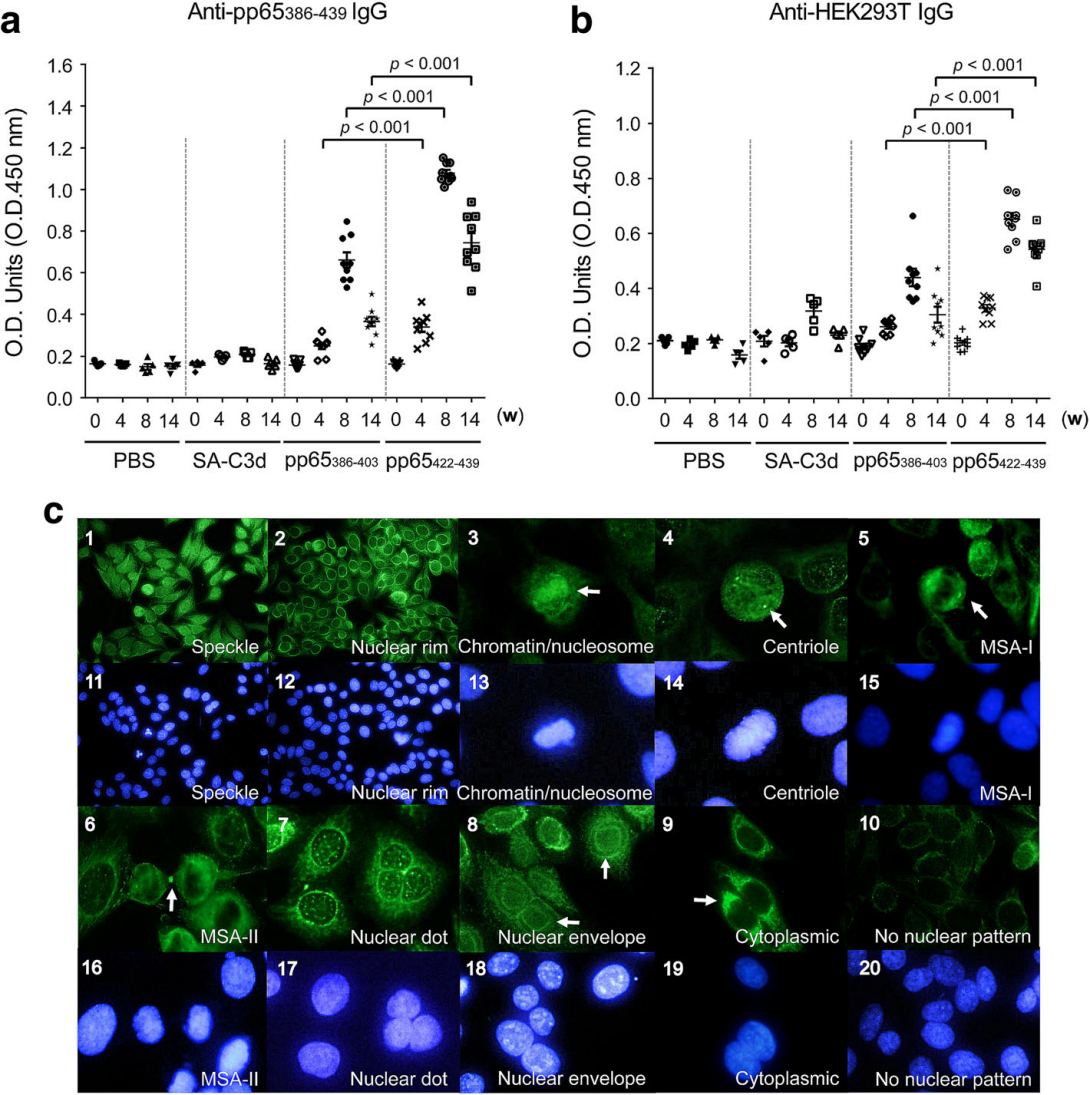
Detection of anti-pp65 and anti-nuclear reactivity from pp65_386-403_, pp65_422-439_, streptavidin-complement C3 (*SA-C3d*) or PBS-immunized serum. The IgG against pp65_386-439_ and HEK293T extract from pp65_386-403_ (n = 9), pp65_422-439_ (n = 9), SA-C3d (n = 5) or PBS (n = 5)-immunized serum at 0, 4, 8 and 14 weeks post immunization were performed at 1:250 dilution. **a** ELISA for anti-pp65_386-403_ and anti-pp65_422-439_ reactivity against pp65_386-439_ peptide. **b** ELISA for anti-HEK293T reactivity against total HEK293T lysate. **c** HEp-2 substrate slides were used for detection of anti-nuclear antibodies. Serum 8 weeks post immunization was 100× diluted for anti-nuclear antibodies (ANA) stain. Nuclear patterns of speckle (*b1*, *b11*), nuclear rim (*b2*, *b12*), chromatin/nucleosome (*b3*, *b13*), centriole (*b4*, *b14*), mitotic spindle type I (*MSA-I*) (*b5*, *b15*), MSA-II (*b6*, *b16*), nuclear dot (*b7*, *b1*7) and nuclear envelope (*b8*, *b18*) were revealed in serum from pp65_422-439_-immunized mice. Cytoplasmic response (*b9*, *b19*) was detected in serum from pp65_386-403_-immunized and pp65_422-439_-immunized mice. Nuclear reactivity was not found in PBS, SA-C3d or pp65_386-403_-immunized mice (*b10*, *b20*). *White arrowheads* indicate the patterns of nuclear or cytoplasmic response. Data are presented as the mean ± SEM of three independent experiments. *w* weeks post immunization, *O.D.* optical density

To evaluate the induction of autoantibodies following exposure of pp65_422-439_, BALB/c mice were immunized with pp65_422-439_, pp65_386-403_, streptavidin (SA) or PBS, using mouse C3d as a molecular adjuvant to improve the immunogenicity of these peptides through CR2-C3d interaction [22, 25]. Both pp65_422-439_ and pp65_386-403_ immunization induced anti-pp65_386-439_-reactive IgG at 4 weeks post immunization and continued until the completion of the observation period (14 weeks post immunization). Quasi-quantitative analysis showed that the pp65_422-439_ induced twice as much of pp65_386-439_-specific antibody titers than pp65_386-403_. The anti-pp65_386-439_ reactive IgG was not detected from either SA-C3d or PBS immunized mice (Fig. 3a and Additional file 2: Figure S2).

The serum from pp65 epitope immunization was tested against HEK293T cell lysate. The immunization of pp65_422-439_ or pp65_386-403_ elicited anti-HEK293T IgG, which was first detected at 6 weeks (data not shown), peaked at 8 weeks and was sustained until 14 weeks post immunization. The pp65_422-439_ immunization (0.667 ± 0.027) induced higher titers than pp65_386-403_ immunization (0.447 ± 0.034, *p* < 0.001) at 8 and 14 weeks post immunization (Fig. 3b). Multiple ANA patterns can be identified following immunization of pp65_422-439_ (Fig. 3c) including speckle (Fig. 3c1, c11), nuclear rim (Fig. 3c2, c12), chromatin/nucleosome (Fig. 3c3, c13), centrioles (Fig. 3c4, c14), MSA I (Fig. 3c5, c15), MSA II (Fig. 3c6, c16), nuclear dots (Fig. 3c7, c17), nuclear envelope (Fig. 3c8, c18) and cytoplasmic proteins stains (Fig. 3c9, c19) at 1:100 dilution 8 weeks post immunization (Additional file 3: Table S1). Immunization with pp65_386-403_ induced a pattern of nuclear dots but it was only detected at 1:40 dilution (data not shown). Nuclear staining was not observed in control mice at 1:40 or higher dilution (Fig. 3c10, c20). Antibodies against cytoplasmic components were found in seven out of nine pp65_422-439_-immunized mice (78%) and three out of nine pp65_386-403_-immunized mice (33%) at 1:100 dilution.

### Immunization of pp65_422-439_ induced high titers of anti-dsDNA antibodies.

**Fig. 4:**
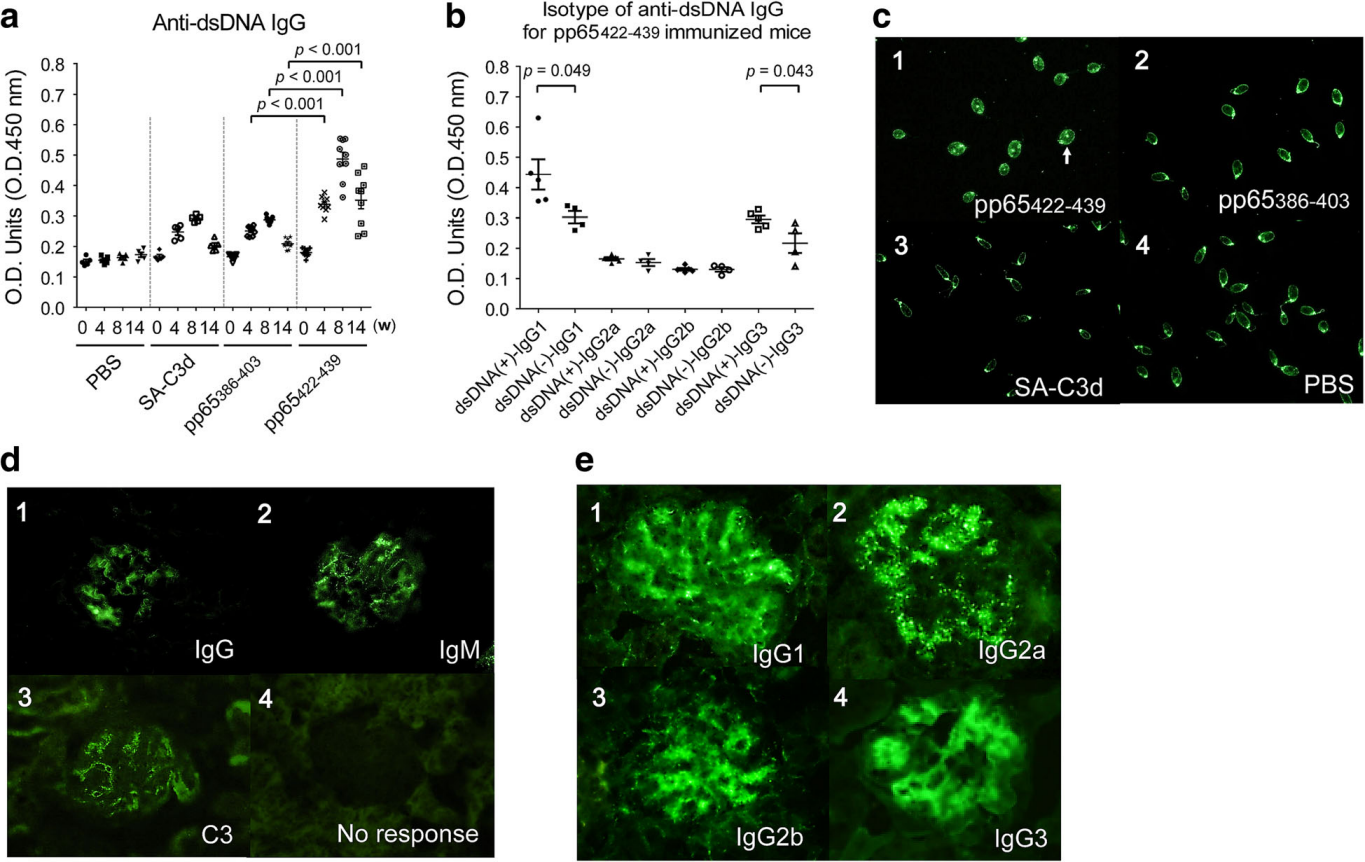
Detection of serum anti-double-stranded DNA (*anti-dsDNA*) antibody and immunoglobulin deposition in glomeruli from immunized mice. **a** ELISA for anti-dsDNA activity with serum at 1:250 dilution. **b** Isotyping of anti-dsDNA antibody for pp65_422-439_ immunized mice (n = 9) at 8 weeks post immunization. Serum was used at 1:250 dilution. *dsDNA(+)* seropositive for dsDNA, *dsDNA(-)* seronegative for dsDNA. **c** Representatives of *C. luciliae* stain by serum from pp65_422-439_ (*c1*), pp65_386-403_ (*c2*), streptavidin-complement 3d (*SA-C3d*) (*c3*) or PBS (*c4*) immunized animals at 8 weeks post immunization at 1:80 dilution. *White arrowheads* indicate dsDNA-positive stains. **d** Kidney sections from pp65_422-439_- immunized mice were stained with fluorescein isothiocyanate (FITC)-conjugated anti-mouse IgG (*d1*), IgM (*d2*) and C3 (*d3*). Immunoglobulin deposition was not observed in glomeruli from SA-C3d-immunized (*d4*) or PBS-immunized mice. **e** Kidney sections from pp65_422-439_-immunized mice were stained with FITC-conjugated anti-mouse IgG1 (*e1*), IgG2a (*e2*) IgG2b (*e3*) and IgG3 (*e4*). Kidneys were collected at 22 weeks of mice age. Data are presented as the mean ± SEM of three independent experiments. *w* weeks post immunization, *O.D.* optical density

Anti-dsDNA antibody is pathognomonic for SLE. To verify the cross-reactivity of anti-pp65_422-439_ antibodies to dsDNA, anti-dsDNA reactivity from immunized animals was tested by both anti-dsDNA ELISA and *C. luciliae* assays. In the ELISA, the pp65_422-439_ immunized mice exhibited significantly higher titers of IgG to dsDNA compared to pp65_386-403_ immunization (Fig. 4a). The anti-dsDNA titers were not elevated in the remaining groups. Isotype analysis of pp65_422-439_ immunization showed that the enhancement of anti-dsDNA is likely contributed by IgG_1_ (dsDNA(+) vs. dsDNA (-), 0.44 ± 0.05 vs. 0.32 ± 0.02, *p* = 0.049) and IgG_3_ (dsDNA(+) vs. dsDNA (-), 0.295 ± 0.01 vs. 0.217 ± 0.03, *p* = 0.043) (Fig. 4b). In the *C. luciliae* stain, three serial dilutions, 1:20, 1:40 and 1:80 were used (Fig. 4c and Table 2). At the lowest dilution (1:20), anti-dsDNA activities were detected from all groups.

**Table 2:** Summary of anti-dsDNA activity in immunized mice

Weeks post immunization	PBS n=5	SA-C3d n=5	pp65_386-403_ n=9	pp65_422-439_ n=9
4	2^w^/5, 2^w^/5, 0/5	3^w^/5, 3^w^/5, 0/5	3^(2,1w)^/9, 3^(1,2w)^/9, 0/9	8/9, 8^(5,3w)^/9, 5^(4,1w)^/9
8	0/5, 0/5, 0/5	0/5, 0/5, 0/5	2^(1,1w)^/9, 1^w^/9, 0/9	9^(8,1w)^/9, 6^(5,1w)^/9, 5/9
14	0/5, 0/5, 0/5	0/5, 0/5, 0/5	0/9, 0/9, 0/9	4^(2,2w)^/9, 3^(2,1w)^/9, 2/9

Mice sera were used at dilution of 1:20, 1:40 or 1:80. W: weak response

The anti-dsDNA activities from controls and pp65_386-403_ gradually disappeared during serial dilutions to 1:40 or 1:80. In contrast, after pp65_422-439_ immunization, 8/9 and 5/9 positive rates were detected at 4 weeks at 1:40 and 1:80 dilutions, respectively. None of the animals with PBS or SA-C3d demonstrated any anti-dsDNA activity at 8 weeks post immunization. One animal with pp65_386-403_ immunization had activity to dsDNA at 1:20 and 1:40 dilutions but this disappeared at 1:80 dilution. In pp65_422-439_ immunization, 9/9, 6/9 and 5/9 anti-dsDNA serum was identified at 1:20, 1:40 and 1:80 dilutions, respectively. At 14 weeks post immunization, all animals except those with pp65_422-439_ immunization had negative dsDNA reactivity. The dsDNA reactivity of pp65_422-439_ immunization at 14 weeks post immunization was reduced to 4/9, 3/9 and 2/9 at 1:20, 1:40 and 1:80 dilutions, respectively.

To study the pathogenicity of pp65_422-439_ reactive antibodies, kidney tissue from immunized mice was examined for signs of immune complex nephritis. Indirect immunofluorescent stains with anti-mouse IgG or IgM revealed that pp65_422-439_-immunized mice developed intense IgG (6/9), IgM (5/9) and C3 (2/9) deposition in the glomeruli (Fig. 4d and Additional file 3: Table S2). In contrast to pp65_422-439_, pp65_386-403_ immunization induced only mild IgM deposition (2/9). No pathological staining was found in PBS (0/5) or SA-C3d immunized mice (0/5). Immunoglobulin isotype deposition in pp65_422-439_-immunized mice revealed that IgG_1_ (6/9) and IgG_3_ (4/9) were dominant isotypes compared to IgG_2a_ (2/9) and IgG_2b_ (1/9) in pp65_386-403_-immunized mice (Fig. 4e).

### The dominant target of HCMV pp65_428-437_ epitope exhibited dsDNA reactivity

**Fig. 5:**
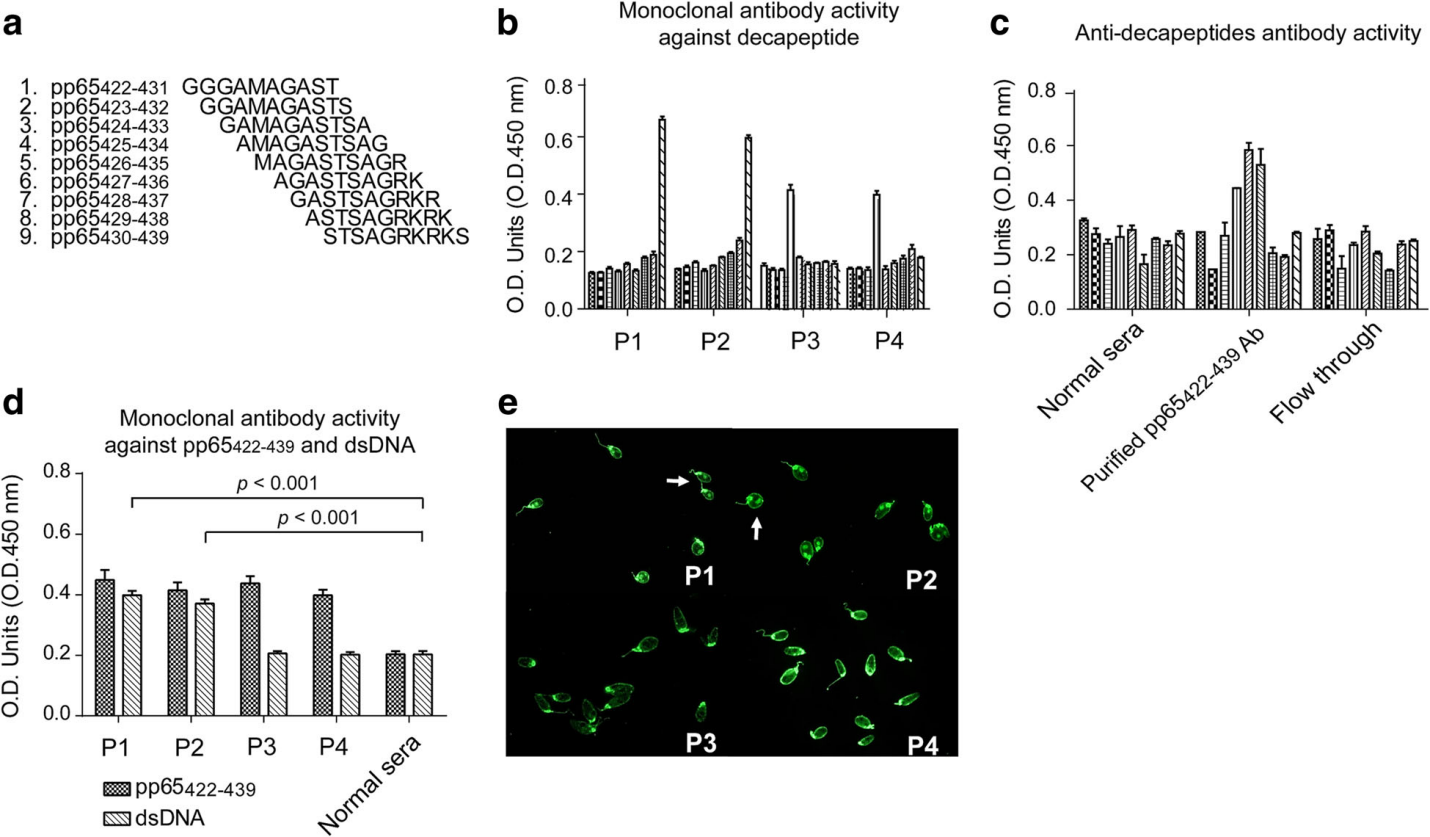
Antibody activity against pp65_422-439_-derived decapeptides and double-stranded DNA (*dsDNA*) from anti-pp65_422-439_ antibodies in human systemic lupus erythematosus (SLE), immunized mice serum and monoclonal antibodies from pp65_422-439_-immunized mice. **a** Representatives of nine overlapping pp65_422-439_-derived decapeptides. Each peptide is shifted by one amino acid. ELISA for anti-decapeptide reactivity with four monoclonal antibodies (**b**) and anti-pp65_422-439_ antibody (**c**) from SLE-dsDNA(+) serum: 100× diluted normal serum and flow-through were used as negative controls. **d** ELISA for IgG against pp65_422-439_ and dsDNA with monoclonal antibodies and 100× diluted normal serum. **e** Representatives of *C. luciliae* stain by P1 (*e1*), P2 (*e2*), P3 (*e3*) or P4 (*e4*) monoclonal antibodies, respectively. *White arrowheads* indicate the positive stains. Data are presented as the mean ± SEM of three independent experiments. *O.D.* optical density

In order to map the B cell epitopes within pp65_422-439_, nine pp65_422-439_-derived decapeptides were synthesized and tested with SLE-dsDNA(+) serum and serum from immunized animals (Fig. 5a). Monoclonal antibodies were also generated from animals immunized with pp65_422-439_. We observed pp65_422-439_-specific reactive monoclonal antibodies (mAb) against pp65_430-439_ (P1, P2) and pp65_425-434_ (P3, P4) (Fig. 5b). The P1 and P2 mAbs also reacted positively in the ELISA and *C. lucilliae* assay (Fig. 5c, d). In human serum assays, pp65_426-437_, which expands three decapeptides, is targeted by anti-pp65_422-439_-specific antibody from SLE-dsDNA(+) (Fig. 5e).

**Fig. 6:**
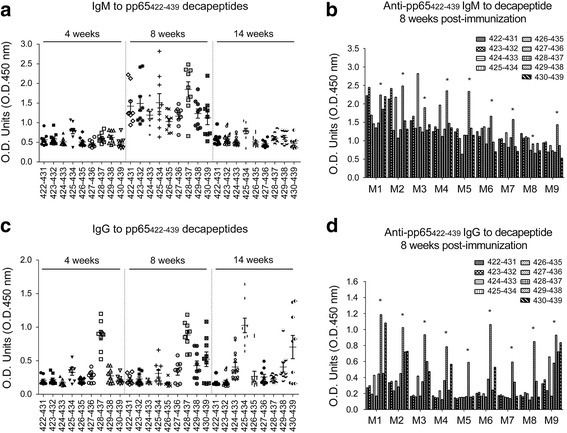
ELISA for IgG/M reactivity against decapeptides from pp65_422-439_-immunized serum. Nine pp65_422-439_ derived decapeptides and 250× diluted mice serum was used. **a** ELISA for IgM against decapeptides from pp65_422-439_-immunized mice (n = 9) at 4, 8 and 14 weeks post immunization. **b** The IgM against nine decapeptides from each pp65_422-439_-immunized mice (n = 9) at 8 weeks post immunization; IgM reactivity to each pp65_422-439_-derived decapeptide. **c** ELISA for IgG against decapeptides from pp65_422-439_-immunized mice (n = 9) at 4, 8 and 14 weeks post immunization. **d** IgG against decapeptides from pp65_422-439_-immunized mice (n = 9) at 8 weeks post immunization: IgG reactivity to each pp65_422-439_-derived decapeptide. *IgM/G against pp65_428-437_. Data are presented as the mean ± SEM of three independent experiments. *O.D.* optical density, *M* mouse

In immunized animals, pp65_422-439_-induced IgM reacted to all decapeptides with elevated titers to pp65_425-434_ at 4 weeks post immunization (Fig. 6a). In addition to pp65_425-434_, few mice also have elevated IgM to pp65_428-437_ and pp65_429-438_. At 8 weeks post immunization, IgM activity in response to decapeptides was enhanced roughly twofold optical density (OD) with the exception of pp65_428-437_, which almost tripled the OD at week 4 (Fig. 6a and b). The IgM activities in response to decapeptides at 14 weeks post immunization were reduced to an OD level similar to week 4. The IgG at 4 weeks post immunization was greatly enhanced in response to pp65_428-437_ in all mice but there was poor response to pp65_425-434_ (Fig. 6c). At 8 weeks post immunization, anti-pp65_428-437_ IgG represented the dominant immune activity, followed by anti-pp65_430-439_ and anti-pp65_429-438_ IgG (Fig. 6d). At 14 weeks post immunization, the IgG activities in response to pp65_430-439_ and pp65_425-434_ were further enhanced and associated with drastic reduction of anti-pp65_428-437_ to its basal level. This reduction of anti-pp65_428-437_ IgG to basal level occurred universally in all of the animals of this group.

## Discussion

Viral peptide-induced autoimmunity in animal models is an emerging field but the underlying mechanisms are not well-understood. Immunization of EBNA-1 or its fragment has been demonstrated to elicit not only immune response to viral antigen, but also IgG activity to 60 kD Ro, SmB/B’ and dsDNA [4, 26, 27]. Herein, we report the high prevalence of serum anti-pp65_422-439_ antibody in patients with SLE. Also, immunization of BALB/c mice with pp65_422-439_-induced cross-reactive autoantibodies against nuclear antigens of host cells, particularly dsDNA, and developed initial signs of nephritis with Ig deposition at 14 weeks post immunization. However, our mapping is unable to completely exclude the possibility that there were discontinuous epitopes, because the B cell epitopes were examined from pp65_386-439_ to pp65_422-439_. The higher incidence of anti-pp65_422-439_ activity in patients with SLE and the instigation of autoimmune-like antibodies through immunization of pp65_422-439_ in BALB/c mice suggested that immunity to pp65_422-439_ might drive pathogenic potential for SLE through epitope spreading and triggering autoantibody production in genetically susceptible individuals.

In our competitive inhibitor assay (Fig. 2b, c), pp65_422-439_ antibody from SLE-dsDNA(+) cross-reactive with dsDNA was not inhibited completely by pp65_422-439_, suggesting that more complex antibody repertoires, for example antibodies that recognize discontinuous epitopes, were obtained from SLE-dsDNA(+) through affinity purification by four tandem-repeats of pp65_422-439_. In addition, pp65_422-439_ antibody from SLE-dsDNA(-) exhibited anti-dsDNA reactivity, but was negative on *C. luciliae* stain. These results were suggested that the anti-dsDNA activity in the ELISA might be due to relatively weak anti-dsDNA reactivity of concentrated anti-pp65_422-439_ antibody from SLE-dsDNA(-). On the other hand, the increase in antibodies to HEK293 and dsDNA observed in SA-C3d-immunized mice at 8 week post-immunization might result from polyclonal B cell activation. However, we did not observe this phenomenon in our analysis of anti-pp65_422-439_ antibody against pp65_386-439_. The absorption and analysis of the B cell repertoire in response to pp65_422-439_ may play a critical part in autoimmunity require further validation.

HCMVpp65 is a well-known T cell antigen in healthy individuals [19, 20]. HCMV pp65 and pp65_336-439_-induced weak humoral responses were verified in healthy humans and BALB/c mice [21, 22]. Unlike normal or other disease controls, anti-pp65_422-439_ antibody occurs more frequently and has higher specificity in patients with SLE, particularly in anti-dsDNA-positive patients. Elevated anti-pp65_336-439_ antibody titers were measured in patients with SLE, but there was no statistically significant relationship between anti-pp65_336-439_ reactivity and serum dsDNA antibody [22].

These observations imply that pp65_422-439_ may contain one more representative epitope, which is associated with the production of anti-dsDNA antibody. Regarding the improvement of immunogenicity of pp65 peptides in the BALB/c model, mouse C3d acts as molecular adjuvant for interplay between innate and adaptive immunity [25]. Immunization of truncated pp65_336-439_ attached to C3d has been demonstrated to induce the development of autoimmunity [22]. In contrast, complete Freund’s adjuvant alone was unable to elicit chronic autoimmunity (data not shown). Immunization of pp65_422-439_ with C3d to BALB/c mice was sufficient to induce anti-pp65_422-439_ antibody. The transient humoral response to pp65_422-439_, also observed in pp65_336-439_-immunized BALB/c mice, indicates that genetic background plays a vital role in exacerbation of SLE.

Anti-dsDNA antibody has served as a critical immunological biomarker and diagnostic criterion for SLE [23, 24]. BALB/c mice challenged by a surrogate peptide have been reported to induce anti-dsDNA antibodies [14]. The nephritogenicity of anti-dsDNA antibody has been shown to mediate cross-reactivity to alpha actinin and annexin II [28, 29]. Also, lupus autoantibodies binding to DNA/nucleosome fragments released from apoptotic cells were observed in the glomerular matrix [30]. Immunization using pp65 or its truncated form has been previously shown to induce multiple anti-nuclear antibodies and anti-dsDNA antibody in BALB/c mice [22]. As expected, anti-dsDNA serum from patients with SLE had anti-pp65 reactivity, particularly to the pp65_422-439_ region. Notably, patients with SLE were double positive to pp65, and simultaneously dsDNA chromatin/nucleosome stain was positive. The anti-pp65 antibody that reacted to dsDNA and chromatin/nucleosome was previously verified in animals immunized for pp65_336-439_ [22].

It has been suggested that anti-nucleosome antibodies are sensitive and specific for lupus nephropathy and the correlation of the antibody titers represent a better biomarker of SLE global disease activity [31, 32]. These consistent results of human and animal studies imply that pp65_422-439_ peptide may possess one critical epitope contributing to the development of SLE. However, the limitations of the present study using stored serum from a cross-sectional study require future study to document their clinical associations with lupus nephropathy and the SLE disease activity damage index.

Following immunization of pp65_422-439_, antigen-specific IgG and IgM were analyzed at 4, 8 and 14 weeks post immunization. This pp65_422-439_ immunization scheme elicited antibodies reactive against antigens from HEK293T cells and produced ANA stain patterns resembling those found in anti-pp65_422-439_-purified antibody stains from patients with SLE. The appearance of autoantibodies in patients with SLE is an indicator of subsequent lupus disease onset [33]. The anti-dsDNA antibodies play critical roles in lupus nephritis; however, elevation of autoantibodies, particularly anti-dsDNA antibodies, has been identified in double-transgenic BALB/c mice expressing both the R4A-gamma2b heavy chain and the anti-apoptotic bcl-2 gene, but the mice did not develop nephritis [34]. In the current study ELISA and the *C. luciliae* assay demonstrated anti-dsDNA reactivity to pp65-purified human antibodies and pp65_422-439_-immunized serum. The pp65_422-439_ immunization scheme not only elicited anti-dsDNA antibodies, but also initiated early-phase kidney damage in BALB/c mice. In the near future, we speculate that pp65_422-439_ reactivity in combination with anti-chromatin/nucleosome and dsDNA antibodies may better fit as a surrogate biomarker of lupus nephropathy inflammation and damage [35, 36].

On terms IgG isotype analysis, both dsDNA and pp65_422-439_-specific IgG were detected in serum from immunized animals, with IgG_1_ and IgG_3_ isotypes. Mouse IgG_3_ is involved in the pathogenic autoimmunity, especially immune complex depositions and glomerulonephritis [37]. IgG_3_ production has been proposed as a critical factor in nephritis among MRL/lpr mice [38]. Similar to human IgG_2,_ T-cell-independent mouse IgG_3_ mainly recognizes carbohydrate epitopes [39]. Human IgG_1_ and IgG_2_ isotypes of anti-nucleohistone and anti-dsDNA antibodies are the predominant isotypes found in plasma from patients with lupus who have renal disease [40]. In pp65_422-439_ immunization, elevated serum titers of anti-dsDNA IgG_1_ and IgG_3_ antibodies positively correlated with the severity of immunoglobulin deposition in glomeruli. Nevertheless, the current study did not provide sufficient evidence to fully explain the causal relationship between pp65-induced anti-dsDNA antibodies and nephritis development in BALB/c mice. The role of pp65_422-439_-induced autoantibodies in glomerular injury required verification by further study.

Three dominant immunological epitopes, pp65_425-434_, pp65_428-437_ and pp65_430-439_, elicited IgG and/or IgM activities at different immunological stages. In the first 4 weeks of immunization, IgG was targeting pp65_428-437_. By 8 weeks post immunization, IgG reacted to pp65_425-434_, pp65_428-437_ and pp65_430-439_, likely as a consequence of epitope spreading. After 14 weeks post immunization, IgG remained active in response to pp65_425-434_ and pp65_430-439_ but lost its activity in response to pp65_428-437_. These findings correlated with our mAb, which had reactivity to pp65_425-434_ and pp65_430-439_. The positive response of mAb P1 and P2 to both dsDNA and pp65_430-439_ suggests that pp65_430-439_ may contain elements that induce the anti-dsDNA response. The anti-dsDNA IgG activities were detected at 4 weeks post immunization with pp65_425-434_ and pp65_428-437_. As mAb P3 and P4 did not possess anti-dsDNA activity, this implies a strong association between anti-pp65_428-437_ and anti-dsDNA activity. Moreover, the anti-pp65_428-437_ activity was well-aligned with anti-dsDNA responses, as seen at weeks 4, 8 and 14 post immunization (Table 2). In humans, pp65_428-437_ is a target for pp65 and dsDNA-specific serum from patients with SLE. These findings suggest that pp65_428-437_ is a potential candidate epitope for promoting anti-dsDNA responses.

The issues of possible factors involved in molecular mimicry and epitope spreading have been widely discussed. The specific amino acid residues interacting with DNA, arginine (R), asparagine (N) and lysine (K), from either virus or necrotic cells, for somatic mutation, occurred during clonal expansion supports the hypothesis that peptide antigen has the potential to elicit the generation of anti-dsDNA antibody [15]. The amino acid 428-439, ASTSAGRKRKSA, of pp65 may contain one hot spot to provoke anti-dsDNA antibody production. However, this hypothesis cannot fully explain the discrepancy in the function of anti-pp65_422-439_ antibodies in dsDNA-positive and dsDNA-negative patients with SLE. We speculate that genetic background bias and preference of major histocompatibility complex (MHC) presentation may be together implicated in autoantibody production and subsequent SLE development.

Over the past few decades, study of HCMV has focused on the high-passage HCMV strain Towne, and AD169, and research into their potential capacity through efficient replication in human fibroblasts. In HCMV infection, pp65 is transported into the nucleus immediately through two nuclear localization sequences, pp65_418-438_ and pp65_537-561_ [41]. The binding of pp65 to metaphase-arrested chromosomes in pp65-expressing fibroblasts during virus infection implies that pp65 may not bind to host proteins, but also forms immune-complex to genetic materials and nuclear components [42]. The SV40 large T-antigen of human polyomaviruses has been demonstrated to form a T-antigen/nucleosome complex, subsequently targeted by host immune responses and accelerates the generation of cross-reactive antibodies against both virus and host during viral replication [43]. Therefore, full-length or fragmented pp65 binding to immune-complexes formed from nuclear binding proteins may not only be targeted by antiviral antibodies but also increase the opportunity for B cell epitope spreading and lead to autoimmunity in genetically susceptible individuals. It is worth mentioning that pp65 shares high homology among different HCMV strains and the fragment of pp65_428-437_, GASTSAGRKR, is highly conserved in HCMV strains such as Towne (pp65_418-427_), AD169 (pp65_428-437_) and Toledo (pp65_428-437_).

In patients with SLE, dsDNA-reactive IgM has been proposed as a protective mechanism that ameliorates autoimmunity and exhibits a negative association with lupus nephritis [44]. Up to now, three possible hypotheses have been proposed to explain how IgM antibody modulates autoimmunity. First, the elevated titer of IgM antibody acts as a competitive role binding to circulating antigens to decrease the formation of the IgG immune complex [45]. Second, IgM antibody downregulates autoreactive B cells to reduce the secretion of pathogenic IgG antibody [46]. Third, the uptake of IgM immune complex by phagocytic cells is more effective in preventing glomerular deposition of immune complex [47]. In pp65_422-439_ immunization, after immunization IgM initially targets the entire pp65_422-439_ with elevated titers to pp65_425-434_. Elevation of IgM to pp65_428-437_ at 8 weeks post immunization was detected after major elevation of IgG response to the same epitope. The IgM response to pp65_428-437_ is linked to anti-dsDNA activities (Additional file 4: Figure S3). However, after autoreactive anti-pp65_428-437_ IgG production, the upregulated IgM subsequently reduced anti-pp65_428-437_ IgG levels, suggesting that pp65_428-437_-specific IgM may be involved in alleviating the autoimmune response through the immune system in the non-autoimmune strain of BALB/c mice. More studies are needed to test the correlation between different classes of Ig and immune responses to specific autoantigens.

## Conclusions

In conclusion, we report here that following immunization of HCMV pp65_422-439_, which is an 18-amino-acid peptide, non-autoimmune-prone animals developed autoimmunity, and exhibited autoantibodies to nuclear components and early signs of nephritis that resemble human SLE. The epitope pp65_428-437_ is the most likely candidate to trigger autoimmunity. The occurrence of epitope spreading in HCMV infection may be a driving force to induce cross-reactive autoantibodies in individuals with genetic predisposition. The amelioration of autoimmunity after elevated production of IgM targeting pp65_428-437_ may be ascribed to the modulation of pathogenic autoreactive IgG response.

## Additional files

Additional file 1: Figure S1. Detection of IgG against pp65_386-439_ in serum from patients with SLE, AS, RA and normal controls. (PDF 2112 kb)
Additional file 2: Figure S2. Detection of anti-pp65 reactivity from pp65_386-403_, pp65_422-439_, SA-C3d and PBS immunized serum. (PDF 1144 kb)
Additional file 3: Summary of ANA patterns in mice against cellular components and isotypes of antibody deposition on glomeruli. (PDF 53 kb)
Additional file 4: Figure S3. Detection of IgM anti-dsDNA antibodies in PBS, SA-C3d, pp65_386-403_ and pp65_422-439_ immunized sera. (PDF 773 kb)

## Abbreviations

ANAs: Anti-nuclear antibodies
C3d: Complement 3d
CFA: Complete Freund’s adjuvant
CnBr: Cyanogen bromide
DAPI: 4',6-Diamidino-2-phenylindole
dsDNA: Double-stranded DNA
EBNA-1: Epstein-Barr virus nuclear antigen 1
EBV: Epstein-Barr virus
ELISA: Enzyme-linked immunosorbent assay
FITC: Fluorescein isothiocyanate
HCMV: Human cytomegalovirus
HRP: Horseradish peroxidase
IFA: Incomplete Freund’s adjuvant
kDa: KiloDalton
mAb: Monoclonal antibodies
MSA-I/II: Mitotic spindle type I/II
OPD: O-phenylenediamine dihydrochloride
PBS: Phosphate-buffered saline
pp65: Phosphoprotein 65
RA: Rheumatoid arthritis
RPMI: Roswell Park Memorial Institute medium
RT: Room temperature
SA: Streptavidin
SLE: Systemic lupus erythematosus
SLE-dsDNA(-): systemic lupus erythematosus without dsDNA antibody
SLE-dsDNA(+): systemic lupus erythematosus with dsDNA antibody
SmB/B’: Smith antigen B/B’
snRNP: Small nuclear ribonucleoprotein
