# Supplementary material for: B cell epitope of human cytomegalovirus phosphoprotein 65 (HCMV pp65) induced anti-dsDNA antibody in BALB/c mice
Source: Arthritis Res Ther. 2017 Mar 21;19:65. doi: 10.1186/s13075-017-1268-2 (PMC5359867; doi:10.1186/s13075-017-1268-2)
Supplement: Additional file 1: Figure S1. — Detection of IgG against pp65386-439 in serum from patients with SLE, AS, RA and normal controls. (PDF 2112 kb) [file 13075_2017_1268_MOESM1_ESM.pdf]

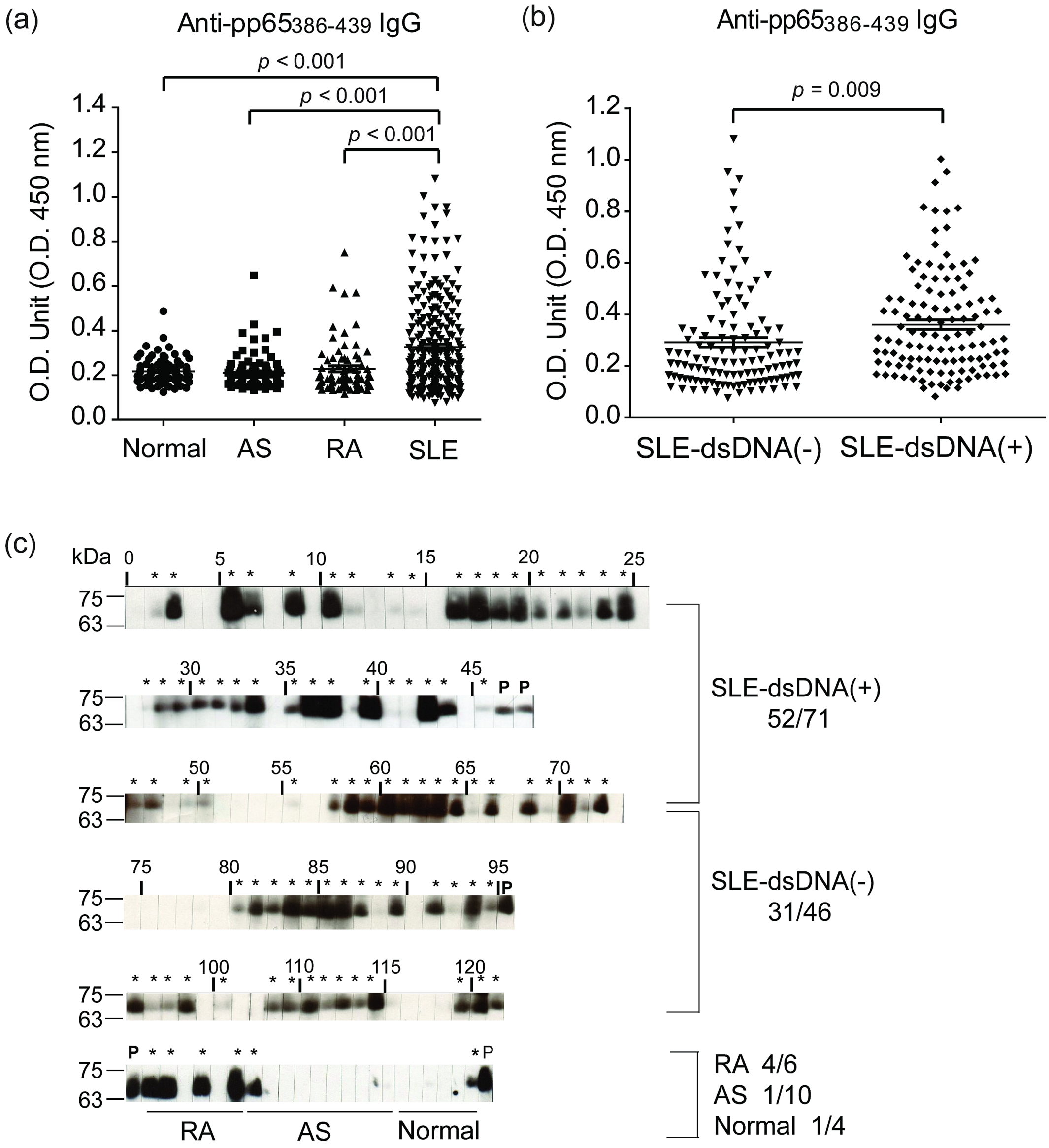

Additional file 1. Detection of IgG against $pp65_{386-439}$ from sera of SLE, AS, RA and normal control. (a) ELISA assays for IgG against $pp65_{386-439}$ with sera from SLE (*n*=238), AS (*n*=86), RA (*n*=78) and normal healthy (*n*=84). 1μg/well $pp65_{386-439}$ and 250x diluted human sera were used in tests. (b) The IgG against $pp65_{386-439}$ with sera from SLE-dsDNA(+) and SLE-dsDNA(-)(0.361 ± 0.018 vs. 0.292 ± 0.019, $P = 0.009$). (c) Reconfirmation of $pp65_{386-439}$ seropositive sera by western blotting. Full-length pp65 antigen (40 μg/per slab gel) and 1000x diluted sera were used for tests. In the ELISA assays, the positivity was defined by mean + 3xSEM of normal sera. OD_{450} >0.250 was considered to be positive. ELISA-based results revealed that 71 SLE-dsDNA(+), 46 SLE-dsDNA(-), 10 AS, 6 RA, and 4 normal healthy were positive for $pp65_{386-439}$. Among them, 52 SLE-dsDNA(+) (52/119, 43.70%), 31 SLE-dsDNA(-) (31/119, 26.05%), 1 AS (1/86, 1.16%), 4 RA (4/78, 5.13%) and 1 normal healthy (1/84, 1.19%) exhibited anti-pp65 reactivity in western blotting results, which are consistent to the anti-$pp65_{386-439}$ antibody titers examined in ELISA tests. Star marks (*) indicate sera are positive for pp65. Molecular mass markers (kD) are shown on the left. MW: molecular weight. kDa: kilodalton. Data are presented as the mean ± SEM of three independent experiments
