# Supplementary material for: B cell epitope of human cytomegalovirus phosphoprotein 65 (HCMV pp65) induced anti-dsDNA antibody in BALB/c mice
Source: Arthritis Res Ther. 2017 Mar 21;19:65. doi: 10.1186/s13075-017-1268-2 (PMC5359867; doi:10.1186/s13075-017-1268-2)
Supplement: Additional file 2: Figure S2. — Detection of anti-pp65 reactivity from pp65386-403, pp65422-439, SA-C3d and PBS immunized serum. (PDF 1144 kb) [file 13075_2017_1268_MOESM2_ESM.pdf]

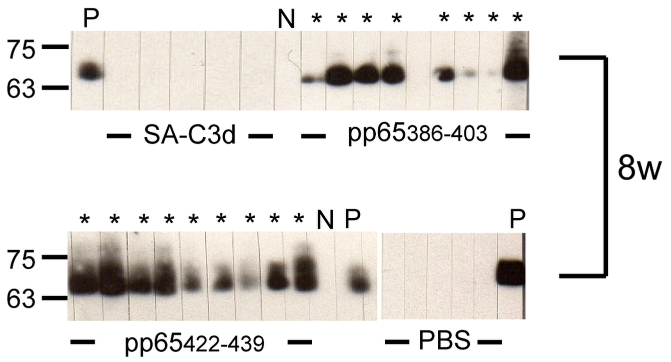

Additional file 2. Detection of anti-pp65 reactivity from pp65$_{386\text{-}403}$, pp65$_{422\text{-}439}$, SA-C3d and PBS immunized sera. Immunoblot assay with sera from pp65$_{386\text{-}403}$ (*n*=9), pp65$_{422\text{-}439}$ (*n*=9), SA-C3d (*n*=5) and PBS (*n*=5) immunized mice against full-length pp65 at 8 weeks post immunization. Sera were used for tests at 1:250 dilution. P: positive control, 5000x diluted Rabbit anti-His tag antibody, N: negative control, 100x diluted normal serum. Secondary antibody: HRP conjugated anti-mouse/rabbit/human IgG antibody.
