# Supplementary material for: B cell epitope of human cytomegalovirus phosphoprotein 65 (HCMV pp65) induced anti-dsDNA antibody in BALB/c mice
Source: Arthritis Res Ther. 2017 Mar 21;19:65. doi: 10.1186/s13075-017-1268-2 (PMC5359867; doi:10.1186/s13075-017-1268-2)
Supplement: Additional file 3: — Summary of ANA patterns in mice against cellular components and isotypes of antibody deposition on glomeruli. (PDF 53 kb) [file 13075_2017_1268_MOESM3_ESM.pdf]

Summary of ANA patterns in mice against cellular components

Nuclear pattern	PBS<br>*n*=5	SA-C3d<br>*n*=5	$pp65_{386-403}$<br>*n*=9	$pp65_{422-439}$<br>*n*=9
Centriole	0	0	0	5
Cytoplasmic proteins	0	0	3	7
MSA I	0	0	0	6
MSA II	0	0	0	7
Nuclear dots	0	0	0	3
Nuclear envelope	0	0	0	3
Nuclear rim	0	0	0	4
Nucleosome/chromatin	0	0	0	4
Speckled pattern	0	0	0	6

MSA I: mitotic spindle type I; MSA II: mitotic spindle type II.

Summary of isotypes of antibody deposition on glomeruli

Antibody isotypes	PBS<br>*n*=5	SA-C3d<br>*n*=5	$pp65_{386-403}$<br>*n*=9	$pp65_{422-439}$<br>*n*=9
IgG+IgM	0	0	0	4
IgM alone	0	0	2	1
IgG alone	0	0	0	2
All negative	5	5	7	2

IgG subclasses	$pp65_{422-439}$
IgG_1+IgG_{2a}+IgG_3	2
IgG_1+IgG_{2b}+IgG_3	1
IgG_1+IgG_3	1
IgG_1	2

These results are representative of triplicated experiments.
