# Supplementary material for: B cell epitope of human cytomegalovirus phosphoprotein 65 (HCMV pp65) induced anti-dsDNA antibody in BALB/c mice
Source: Arthritis Res Ther. 2017 Mar 21;19:65. doi: 10.1186/s13075-017-1268-2 (PMC5359867; doi:10.1186/s13075-017-1268-2)
Supplement: Additional file 4: Figure S3. — Detection of IgM anti-dsDNA antibodies in PBS, SA-C3d, pp65386-403 and pp65422-439 immunized sera. (PDF 773 kb) [file 13075_2017_1268_MOESM4_ESM.pdf]

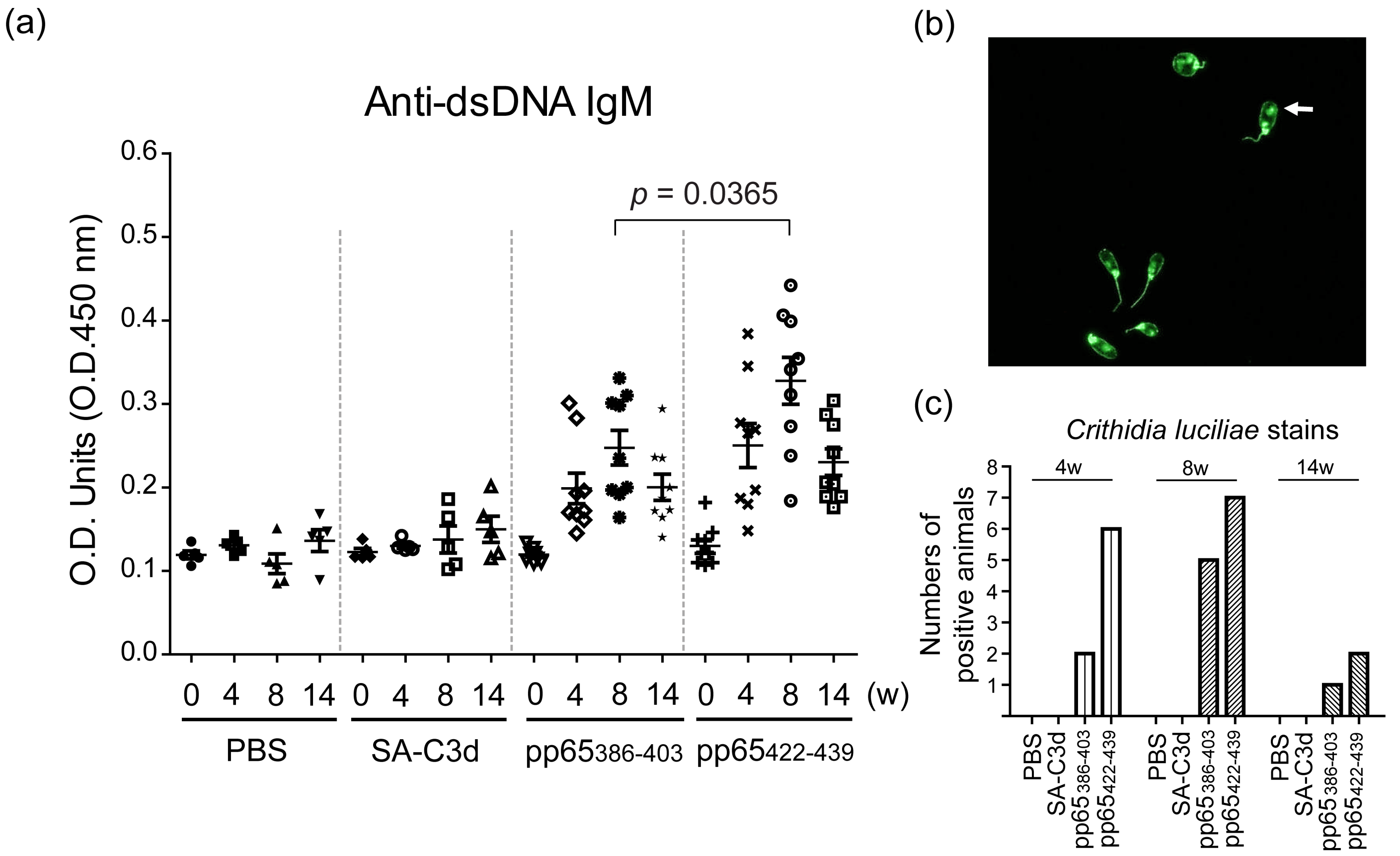

Additional file 4. Detection of IgM anti-dsDNA antibody in PBS, SA-C3d, pp65$_{386-403}$ and pp65$_{422-439}$ immunized sera. (a) ELISA assay for IgM anti-dsDNA antibody activity from pp65$_{422-439}$ (*n*=9), pp65$_{386-403}$ (*n*=9), SA-C3d (*n*=5) and PBS (*n*=5) immunized mice sera. 250x diluted sera were used in ELISA assays for anti-dsDNA reactivity. (b) Representatives of *Crithidia luciliae* stain by sera from pp65$_{422-439}$ mice sera at dilution of 1:80. White arrowheads indicate dsDNA positive stains. (C) Numbers of *Crithidia luciliae* positive animals at 4, 8 and 14 weeks post immunization. These results are representative of triplicated experiments. Data are presented as the mean ± SEM of three independent experiments

.
